# Contactless Cardiovascular Assessment by Imaging Photoplethysmography: A Comparison with Wearable Monitoring

**DOI:** 10.3390/s23031505

**Published:** 2023-01-29

**Authors:** Valerie A. A. van Es, Richard G. P. Lopata, Enzo Pasquale Scilingo, Mimma Nardelli

**Affiliations:** 1 Department of Biomedical Engineering, University of Technology, P.O. Box 513, 5600 Eindhoven, The Netherlands; 2 Bioengineering and Robotics Research Centre E. Piaggio, Dipartimento di Ingegneria dell’Informazione, University of Pisa, Largo Lucio Lazzarino 1, 56122 Pisa, Italy

**Keywords:** imaging photoplethysmography (iPPG), pulse rate variability (PRV), remote photoplethysmography, autonomic nervous system (ANS)

## Abstract

Despite the notable recent developments in the field of remote photoplethysmography (rPPG), extracting a reliable pulse rate variability (PRV) signal still remains a challenge. In this study, eight image-based photoplethysmography (iPPG) extraction methods (GRD, AGRD, PCA, ICA, LE, SPE, CHROM, and POS) were compared in terms of pulse rate (PR) and PRV features. The algorithms were made robust for motion and illumination artifacts by using ad hoc pre- and postprocessing steps. Then, they were systematically tested on the public dataset UBFC-RPPG, containing data from 42 subjects sitting in front of a webcam (30 fps) while playing a time-sensitive mathematical game. The performances of the algorithms were evaluated by statistically comparing iPPG-based and finger-PPG-based PR and PRV features in terms of Spearman’s correlation coefficient, normalized root mean square error (NRMSE), and Bland–Altman analysis. The study revealed POS and CHROM techniques to be the most robust for PR estimation and the assessment of overall autonomic nervous system (ANS) dynamics by using PRV features in time and frequency domains. Furthermore, we demonstrated that a reliable characterization of the vagal tone is made possible by computing the Poincaré map of PRV series derived from the POS and CHROM methods. This study supports the use of iPPG systems as promising tools to obtain clinically useful and specific information about ANS dynamics.

## 1. Introduction

Heart rate (HR) and heart rate variability (HRV) are crucial markers of an individual’s psychophysical status. The HR is the mean number of heartbeats in a time interval, generally considered over one minute, e.g., beats per minute (BPM), whereas HRV refers to the fluctuations in the duration of the time intervals between consecutive heartbeats and reflects the ability of the autonomic nervous system (ANS) to maintain homeostasis. Alterations in HR are completely nonspecific and can be observed in many physiological and pathological conditions, including sleep, physical exercise, stress, anxiety, illness, and assumptions about drugs [1]. On the other hand, the analysis of HRV represents a useful tool for the overall characterization of autonomic dynamics and can provide useful information about alterations in vagal activity [2].

The state-of-the-art method to extract HRV is based on the acquisition of electrocardiographic (ECG) signals, placing multiple electrodes on specific anatomical body positions. The wearable monitoring devices based on photoplethysmography (PPG) do not require this constraint. PPG techniques rely on noninvasive optical sensors applied in contact with the skin to measure the change in the cardiac blood volume pulse (BVP) (for example, from the finger, wrist or ear of the subject) [3]. The BVP signal is obtained by measuring the variations of reflected light in the peripheral skin tissues: the amount of light that reflects by penetrating the skin provides an indication of the changes in blood flow [4]. The pulsatile component of the PPG signal oscillates with the heart cycle period, and thus, the functional characteristics of pulse rate (PR) and pulse rate variability (PRV) extracted from the PPG signal are comparable to the HR and HRV extracted from the ECG [3,4]. Even if PPG sensors have several benefits compared with the ECG sensors (ease of use, low costs, convenience, etc.), they still need to be applied directly to the skin, constraining their feasibility in situations where free mobility is required, in presence of skin damage (burn/ulcer/trauma), or when sensors are too obtrusive, for example, during the monitoring of preterm babies. Noncontact measurement of PR and PRV would be a potential solution for these practical issues, simplifying data acquisition in nonclinical scenarios (e.g., human–machine interaction [5], driver monitoring [6]), and allowing effective remote monitoring of patients [7]. Studies on contactless measurements of PR and PRV include thermal imaging [8], microwave Doppler effect [9], PR from speech [10], and image-based photoplethysmography (iPPG) [11]. In this study, we focused on PR and PRV extraction by making use of the remote-camera-based iPPG method. In addition to the fact that the use of wearable sensors can be avoided using iPPG, the technique is easily scalable, since digital cameras are inexpensive and widely available. The iPPG method is based on similar principles as the PPG optical sensors. The initiated pulse wave travels through the arterial vascular system reaching various parts of the body and resolves a short-termed change in blood volume in the observed skin region. In the skin region, the intensity of the absorbed light depends on the blood volume [12]. So, due to changes in the RGB channel intensities, the BVP can be estimated. For the iPPG signal acquisition, video sequences are usually taken from the subject’s face due to the high blood supply and imaging simplicity.

In the recent scientific literature, several image processing methods have been proposed for the extraction of BVP signals from face videos [13,14,15]. The continuous and rapid development of iPPG extraction methods emphasizes the importance of comparing different methods for iPPG-based PR and PRV estimation [16].

In this framework, we compared the following iPPG extraction methods: the green–red difference (GRD) method [17]; the adaptive green–red difference (AGRD) [18]; the principal component analysis (PCA) [19]; the Laplacian eigenmap (LE) [20]; the stochastic proximity embedding (SPE) [21]; the independent component analysis (ICA) [22]; the chrominace-based (CHROM) method [23]; and the plane-orthogonal-to-skin (POS) method [13]. A short description of the eight methods compared in this study is reported in Table 1.

Theoretical comparison can be complicated because iPPG extraction algorithms consist of multiple steps that are often nonuniformly described. To overcome this complexity, the proposed main steps of iPPG-based PR estimation according to [15] were applied. In addition to their main steps, a stepwise overview of the PRV extraction was included.

The publicly available UBFC-RPPG dataset designed for iPPG analysis was used to test the performances of the eight algorithms [24]. Considering that the UBFC-RPPG dataset mimics realistic computer interaction including rigid-and nonrigid face movements and illumination variances, we aimed at optimizing the algorithms by applying ad hoc pre- and postprocessing steps to create robustness for the movements and illumination variances, and thus, make the image-based PPG applicable for daily use.

To summarize, the general objective of this study is to verify if the iPPG system can successfully provide clinically useful information on ANS regulation, in particular in the field of vagal activity quantification, where the study of HRV (and therefore of PRV) is considered crucial. To this end, we evaluated iPPG reliability in terms of PRV metrics in time, frequency, and nonlinear domains, in addition to the PR mean, which is less sensitive to errors due to its averaging effect and less specific regarding the quantification of ANS modulation. Considering that several different algorithms can be used for iPPG signal extraction, we compared eight methods (see Table 1) to investigate which algorithm shows the best performance in terms of PRV features in realistic human–machine interaction scenarios. Among these methods, SPE was tested for the first time in the field of iPPG signal processing. The iPPG-based PR and PRV metrics were compared with the state-of-the-art wearable technique, the finger clip PPG.

## 2. Materials and Methods

### 2.1. Dataset Description

**Figure 1:**
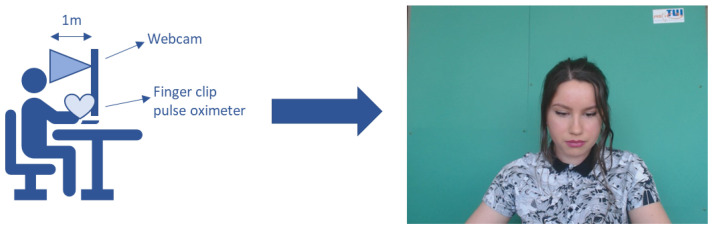
Experimental setup for the data acquisition, including the webcam-based videos to extract the iPPG signal and the ground truth PPG signal acquired using a finger clip pulse oximeter. A frame taken from one of the UBFC-RPPG videos is shown in figure [24].

The UBFC-RPPG dataset [24] consists of 42 videos acquired from 42 subjects. The dataset was created by making use of a custom C++ application for video acquisition while filming using a simple low-cost webcam (Logitech C920 HD Pro) with a resolution of 640 × 480 in uncompressed 8-bit RGB format at 30 fps. All experiments were conducted indoors with a varying amount of sunlight and indoor illumination. The subjects were required to be seated one meter away from the camera while playing a sensitive mathematical game that aimed at an augmentation of the heart rate and simultaneously emulating a normal human–computer scenario. Since the mathematical task required the full focus of the subject, they kept staring at the screen during the experiment. Therefore, the face remained always visible in the webcam-recorded video. To receive the ground truth data, a pulse oximeter finger clip sensor (Contec Medical CMS50E) was used to acquire PPG signals synchronized with each video. The experimental setup and an exemplary frame of one video clip can be seen in Figure 1. The duration of the video recordings in the UBFC-RPPG dataset varies from 45.6 s (1368 frames) to 68.4 s (2052 frames).

The image quality assessment performed on the UBFC-RPPG dataset provided medium averaged values of BRISQUE [25] and PIQE [26] scores (48.544 ± 2.150 and 52.446 ± 6.460, respectively, considering a range from 0 to 100).

### 2.2. iPPG Signal Processing

**Figure 2:**
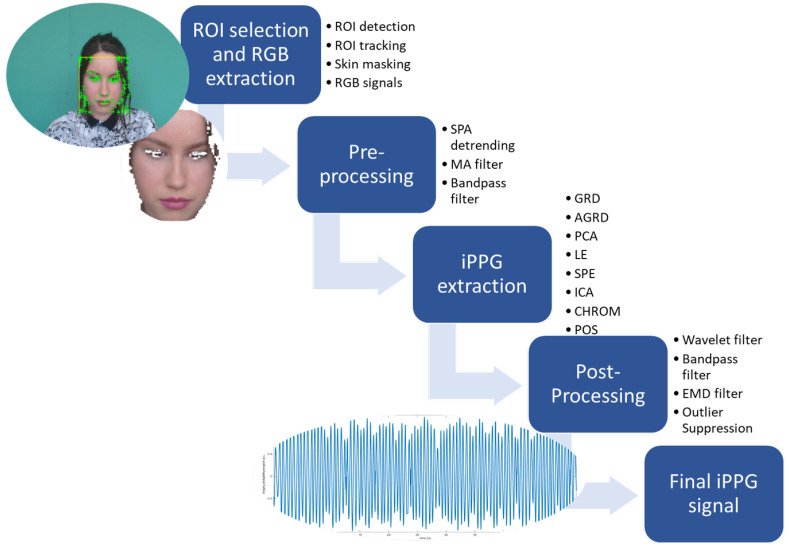
Flow chart of the four steps of blood volume pulse signal extraction using imaging photoplethysmography. A frame taken from one of the UBFC-RPPG videos is shown in the figure [24], the result of face detection is shown in green.

The proposed algorithmic framework for iPPG signal extraction makes use of eight different image-based extraction algorithms (GRD, AGRD, PCA, LE, SPE, ICA, CHROM, and POS), whose performances are optimized through ad hoc signal pre- and postprocessing steps. A sequence of RGB frames is taken as an input. Each frame consists of pixels with coordinates *i,j*, which can be described by vectors $c_{i,j}\left(t\right)={\left(r_{i,j}\left(t\right),g_{i,j}\left(t\right),b_{i,j}\left(t\right)\right)}^{T}$, i.e., the transposed red $r_{i,j}\left(t\right)$, green $g_{i,j}\left(t\right)$, and blue $b_{i,j}\left(t\right)$ channels. The iPPG extraction algorithm comprises four substeps, as presented in the workflow image in Figure 2. To retrieve the final iPPG signal for accurate comparison with the finger-clip-extracted PPG signal, four substeps need to be completed: (1) ROI selection and RGB extraction, where the changing RGB signal in the skin pixels can be measured due to the passing blood pulse; (2) preprocessing, where the raw RGB signal is detrended and filtered to assure blood pulse measurements in a physiological range; (3) iPPG extraction, where the filtered RGB signal is translated in the raw iPPG signal (in this study, this step was executed testing eight different algorithms to allow a performance comparison); and (4) postprocessing, where the raw iPPG signal is removed from noise. When completing this final step, the iPPG signal is ready for PR and PRV analysis. Concerning the second and the last substeps, the selection of the filter processes is based on reported methods in the literature. Then, these methods were experimentally tested on the UBFC-RPPG dataset, where only the pre- and postprocessing method combinations with the best performances were applied. The combination of the SPA detrending with the MA filter and bandpass filter shows the most promising results for the preprocessing of the RGB signal. On the other hand, the combination of the wavelet, bandpass, EMD filters, and the outlier suppression was found to be the most promising for the postprocessing of the initial iPPG signal. Each substep is extensively discussed below.

#### 2.2.1. Region of Interest (ROI) Selection and RGB Extraction

The purpose of this algorithmic step is to constitute the temporal RGB signals that are later used to estimate the iPPG signal. These raw signals are directly extracted from the region of interest (ROI). Selecting a suitable ROI is therefore a crucial and challenging step, as it has a direct impact on the reliability and accuracy of the overall algorithm [27]. The raw RGB signal extraction exists out of four substeps, including face detection, face tracking, skin masking, and raw RGB signal extraction.

1. Face detection: There are three main popular approaches for the ROI choice, namely taking a rectangular ROI encompassing the whole face [11,23,28,29], considering the forehead and cheekbone regions [30,31], or considering only the forehead [32]. Since, in the UBFC-RPPG dataset, hair covers the forehead region for several subjects, our approach considers the whole face region as ROI. To detect the face and select the ROI, a facial rectangle is located for the first frame, employing a cascade classifier constructed using the Viola–Jones algorithm [33].
2. Face tracking: To eliminate the problem of rigid head motions where the subject moves their head outside the defined ROI bounding box, a face tracking system is desirable. Given the high computational cost of the Viola–Jones algorithm, we use the Lucas–Kanase approach (KLT tracker) [34], tracking specific features of the face over time.
3. Skin masking: Only skin pixels contribute to the PR-related information. Therefore, skin masking is performed on every frame to filter out the nonskin pixels. The RGBH-H-CbCr skin color model [35] is applied: a skin color map is determined based on the skin color distribution and utilized on the chrominance segments of the input frames to distinguish pixels that seem to be skin.
4. Raw RGB signal extraction: The time-variant raw RGB signals $c^{0}\left(t\right)=\left(r^{0}\left(t\right),g^{0}\left(t\right),b^{0}\left(t\right)\right)$ are produced by calculating the average pixel value of the skin pixels within the ROI for all frames over time. The average color intensities over the ROI frames in time are calculated as follows:
  (1) $$c^{0}\left(t\right)={\left(r^{0}\left(t\right),g^{0}\left(t\right),b^{0}\left(t\right)\right)}^{T}=\frac{1}{\left|ROI(t)\right|}\sum_{\left(i,j)\epsilon ROI(t\right)}c_{i,j}\left(t\right)$$

#### 2.2.2. Preprocessing of RGB Signals

Noise and artifacts are removed from the raw RGB signals for each frame, making sure to not suppress frequency components in the human heart rate bandwidth. The three preprocessing steps applied in this study are detrending, moving average (MA), and bandpass filtering [36]. Detrending of the raw RGB signal is a substantial step, as the pulsatile component of the iPPG signal has a much lower amplitude than the slowly varying baseline [17]. In this study, the smoothness priors approach (SPA) is applied according to [11,37]. The great advantage of this method over a simple mean-centering detrending method is that the trend can be removed without affecting the PR bandwidth. Applying the moving average (MA) filter, smoothed signals c(t)=(r(t),g(t),b(t)) are obtained, where the high-frequency noise is suppressed. The bandpass filter suppresses frequency components outside the PR bandwidth (0.65–4 Hz) [11,28].

#### 2.2.3. iPPG Signal Extraction

The aim of this step in the algorithm is to extract the raw iPPG signal, i.e., $iPPG^{0}$, computing the weights for each of the preprocessed RGB color signals as follows:

(2) $$iPPG^{0}\left(t\right)=w\left(t\right)\cdot c\left(t\right)=w_{r}\left(t\right)r\left(t\right)+w_{g}\left(t\right)g\left(t\right)+w_{b}\left(t\right)b\left(t\right)$$

where $w_{r}$, $w_{g}$, and $w_{b}$ are, respectively, the weights of the R, G, and B color channel signals [15]. To compute these weights, different methods can be deployed. In this framework, eight different methods are compared: the GRD, AGRD, PCA, ICA, LE, SPE, CHROM, and POS.

⯀ GRD: In the GRD method [17], the green channel is used for the extraction of iPPG, since it generally has the highest signal amplitude [38], whereas the red channel is considered to be an artifact. Thus, the iPPG signal can be computed as follows:
  (3) $$iPPG^{0}\left(t\right)=g\left(t\right)-r\left(t\right)$$
⯀ AGRD: The AGRD method [18] includes an adaptive bandpass filter with the aim to remove residual motion artifacts within the iPPG signal. The approach can be described by the following equation:
  (4) $$iPPG^{0}\left(t\right)=\left|\right|c^{0}\left(t\right)\left|\right|\left(\frac{g(t)}{g^{0}\left(t\right)}-\frac{r(t)}{r^{0}\left(t\right)}\right)$$
  with
  (5) $$\left|\right|c^{0}\left(t\right)\left|\right|=\sqrt{{\left({\left(r^{0}\left(t\right)\right)}^{2}+{\left(g^{0}\left(t\right)\right)}^{2}+{\left(b^{0}\left(t\right)\right)}^{2}\right)}^{2}}$$
  It should be noted that preprocessing is an essential step for the AGRD method because, otherwise, $g\left(t\right)=g^{0}\left(t\right)$ and $r\left(t\right)=r^{0}\left(t\right)$ will result in a zero iPPG signal.
⯀ PCA: The PCA procedure [19,28,39] is a linear dimensionality reduction technique that identifies patterns in the RGB signals in order to capture intensity variations due to blood pulses. In the PCA, a set of observed signals from correlated variables is projected into a linearly uncorrelated orthogonal basis, called principal components. The principal components are defined by $s_{i}=f_{i}^{T}\cdot X$, where *X* is the set of observed signals and $f_{i}$ are the corresponding eigenvectors of the covariance matrix $C=E(XX^{T})$. The number of principal components is usually lower than the number of observed multivariate signals.
⯀ ICA: The ICA technique [11,22,40] is the most popular blind source separation technique for iPPG computation. It is used to separate unknown source signals S(t) from a set of observed mixed signals given by X(t)=AS(t), where A is the mixing matrix [14]. The approximated source signals can be found as $\hat{S}\left(t\right)=Wt$, where *W* is the separation matrix that approximates the inverse of *A*. ICA assumes that the components are statistically independent and non-Gaussian and will then choose the component with the most prominent peak in the PR bandwidth.
⯀ CHROM: The CHROM method, as proposed by [23], aims for robustness to subject motion by employing a model of PPG-induced variations in color intensity. For this technique, the iPPG signal is defined as:
  (6) $$iPPG^{0}\left(t\right)=x_{1}\left(t\right)-\frac{\sigma_{1}\left(t,L\right)}{\sigma_{2}\left(t,L\right)}x_{2}\left(t\right)$$
  with $\sigma_{1}\left(t,L\right)$ and $\sigma_{2}\left(t,L\right)$ being the *L*-point running standard deviations, defined as:
  (7) $$\sigma_{i}\left(t,L\right)=\sqrt{\frac{1}{L-1}\sum_{k=0}^{L-1}{\left(x_{i}\left(t-k\right)\right)}^{2}-\frac{1}{L(L-1)}{\left(\sum_{k=0}^{L-1}x_{i}\left(t-k\right)\right)}^{2}}$$
  for *i* = 1, 2 of $x_{1}\left(t\right)=0.77r\left(t\right)-0.51g\left(t\right)$ and $x_{2}\left(t\right)=0.77r\left(t\right)+0.51g\left(t\right)$. In the algorithm used for this framework, *L* = 1.6 s, as suggested in [23].
⯀ POS: The POS method [13] is quite similar to the CHROM method and can be considered as its simplified and improved version. The iPPG signal is calculated as follows:
  (8) $$iPPG^{0}\left(t\right)=x_{1}\left(t\right)+\frac{\sigma_{1}\left(t,L\right)}{\sigma_{2}\left(t,L\right)}x_{2}\left(t\right)$$
  Here, $\sigma_{1}\left(t,L\right)$ and $\sigma_{2}\left(t,L\right)$ are again the *L*-point running standard deviations of $x_{1}\left(t\right)$ and $x_{2}\left(t\right)$, respectively. However, now, they are defined as $x_{1}\left(t\right)=g\left(t\right)-b\left(t\right)$ and $x_{2}\left(t\right)=g\left(t\right)+b\left(t\right)-2r\left(t\right)$. Like in the CHROM method, *L* corresponds to 1.6 s, as suggested in [13].
⯀ LE: The LE method [20] is a technique aimed at unfolding a nonlinear data distribution in a hyperdimensional space, in order to reduce its dimensionality. When the approach is applied as the iPPG extraction method, it should increase the accuracy in the separation of the iPPG signal from residual sources of fluctuations in light. The LE algorithm maps the averaged RGB signals for the *i*th frame into a three-dimensional (R-G-B) space, and the final goal is to map their distribution onto a one-dimensional space, preserving the local relationship between data points. Firstly, the adjacent graph *G* is constructed, computing the Euclidean distance between the data points. Nodes *i* and *j* of the graph *G* are considered adjacent if *i* is among the *k*-nearest neighbors of *j* and vice versa. Then, manifold learning is used to solve the following optimization problem:
  (9) $$min\sum_{i,j=1}^{n}\left|\right|y_{i}-y_{j}{\left|\right|}^{2}W_{ij}$$
  where $y_{i}$ and $y_{j}$ are two points which are nearest neighbors in the low-dimensional space, and $W_{ij}$ is the weight that measures the closeness of the points $x_{i}$ and $x_{j}$ in the higher-dimensional space. For the extraction of the iPPG signal, the parameter *k* is usually set at 12 [20]. In our study, we tested the LE algorithm using different distance metrics (Euclidean, city-block, Chebyshev, Minkowski, and Mahalanobis) and changing the value of the parameter *k*. According to the experimental results on the UBFC-RPPG dataset, the Mahalanobis distance with *k* = 9 resulted the most promisingly.
⯀ SPE: SPE [21] is a self-organizing algorithm used to produce low-dimensional embeddings that preserve similarities between a set of related observations. In this study, the SPE approach is applied for the first time to RGB data. In our case, the similarities are the fluctuations in the RGB signal intensities due to blood pulsation, from which the iPPG signal can be estimated. The method starts with an initial configuration, and iteratively refines itself by randomly selecting points $x_{a},x_{b}$ and adjusting their coordinates to match the Euclidean distances $d_{a,b}$ on the map more closely to their respective proximities $r_{a,b}$. To avoid oscillatory behavior, the magnitude of the adjustments was controlled by a learning rate parameter, λ, which decreases during the data point refinement. The refined coordinates are updated by:
  (10) $$x_{a}\leftarrow x_{a}+\lambda \frac{1}{2}\frac{r_{ab}-d_{ab}}{d_{ab}+\epsilon }\left(x_{a}-x_{b}\right)$$
  and
  (11) $$x_{b}\leftarrow x_{b}+\lambda \frac{1}{2}\frac{r_{ab}-d_{ab}}{d_{ab}+\epsilon }\left(x_{b}-x_{a}\right)$$
  where ϵ is a small value to avoid the division by zero.

#### 2.2.4. Postprocessing of iPPG Signals

Postprocessing of the photoplethysmographic signal $iPPG^{0}$, extracted from RGB channels, is the fourth and last algorithmic step. Postprocessing improves the quality of the signal and can be especially useful when the artifacts and noise were not (completely) removed at the preprocessing step. Within this framework, the wavelet bandpass filtering, the EMD procedure, and outlier suppression are considered.

1. **Wavelet filtering:** An adaptive two-step wavelet filtering [13,17,18,41] is applied, assuming that frequency components of the $iPPG^{0}$ signal related to noise have weaker power with respect to the components related to the PR. The first step of the method was to perform a continuous wavelet of the $iPPG^{0}$ signal. Here, the wavelet coefficients with a wide Gaussian window centered at a scale corresponding to the maximum of squared wavelet coefficients are averaged over a 15 s temporal running window. Secondly, a general Gaussian filter is applied. To reconstruct the iPPG signal, the inverse continuous wavelet is performed [41].
2. **Empirical mode decomposition:** The purpose of empirical mode decomposition (EMD) [42] is to split the $iPPG^{0}$ signal into a noise component and PR-related component. The EMD technique decomposes the signal into several unique intrinsic mode functions (IMFs) and one residue function (R) according to:
  (12) $$g\left(t\right)=\sum_{i=1}^{n}IMF_{i}\left(t\right)+R_{n}\left(t\right)$$
  with $IMF_{i}\left(t\right)$ the $i^{th}$ IMF at time step *t*, $R_{n}\left(t\right)$ the residue function at time step t, and n the number of EMD iterations. The extracted IMF signal is the filtered main signal, making the peak frequency more lucid and, herewith, making the iPPG signal more reliable for PR and PRV metric extraction.
3. **Outlier suppression:** Am MA filter was again applied to smooth out the signal and suppress the high-frequency peaks that correspond to noise.

**Figure 3:**
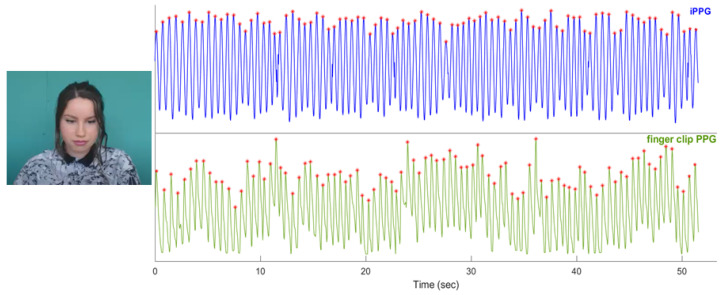
An example of an iPPG signal extracted through the POS method from the video clip of the first subject of the UBFC-RPPG datset (111.6 bpm were found with both iPPG and finger clip PPG monitoring techniques). A frame taken from the video of the first subject of the UBFC-RPPG dataset is shown in the figure [24].

Figure 3 shows the iPPG signal extracted from the video clip of the first subject of the UBFC-RPPG dataset by using the POS method.

### 2.3. Pulse Rate and Pulse Rate Variability Analysis

In this study, we compared cardiovascular metrics extracted from iPPG and finger PPG through the study of PR and PRV. In order to extract such information, the interbeat interval series (IBI) was extracted from both PPG signals, estimating the time intervals between the successive systolic peaks [43,44]. Ectopic beats were corrected by using the Kubios HRV tool, obtaining the series of normal-to-normal (NN) intervals [45]. Each IBI corresponds to a cardiac cycle, and thus, the PR is equal to the inverse of the IBI duration. The average PR is the mean value of PR in the whole signal duration. In order to obtain a comprehensive characterization of the performances of the two techniques (contactless and wearable) for the assessment of cardiovascular dynamics, we analyzed not only the average PR but also the features extracted from the PRV in the time and frequency domains. Concerning the time domain, two statistical indexes were computed: the root mean square of the successive differences (RMSSD) and the standard deviation of NN intervals (SDNN). In addition, we computed two geometrical parameters based on the study of the probability density distribution of NN intervals: the triangular index (TI) and the triangular interpolation of the NN intervals (TINN).

As regards the frequency domain, we considered the power in three main bandwidths: very low frequency (VLF, below 0.04 Hz), low frequency (LF, between 0.04 Hz and 0.15 Hz), and high frequency (HF, between 0.15 and 0.4 Hz). The normalized values of the power in the LF and HF bands were also computed ($LFnu=\frac{LF}{LF+HF}$, $HF=\frac{HF}{LF+HF}$), together with the LF/HF ratio.

Applying chaos theory methodologies to characterize PRV nonlinear dynamics, we studied the Poincaré first return map of the NN interval series. We quantified the point dispersion in the Poincaré map by using the ellipse fitting method [46]. The features SD1 and SD2 represent the standard deviation of the points along the short and long axis of the ellipse that best fits the data in the Poincaré map, and SD1/SD2 is their ratio [46,47].

An overview of the PRV metrics is given in Table 2.

### 2.4. Quality Metrics

To assess the reliability of the estimated PRV features extracted from iPPG, we compared them with the related finger clip PPG reference values. The following statistical analyses were applied: Spearman’s correlation analysis, Bland Altman analysis, and normalized root mean square error computation. The Spearman correlation analysis was applied to quantify the monotonic relationship between the estimated (iPPG-based) and reference (finger clip PPG-based) PR and PRV metrics. The use of such a nonparametric test is justified by the non-Gaussian distribution of the samples (*p* < 0.05 of the null hypothesis of having Gaussian samples of the Kolmogorov–Smirnov test [48]). The Spearman correlation coefficient ρ and the related *p*-values were extracted form each correlation test.

To measure the quality of fit between the estimated (iPPG-based) and the reference (finger clip PPG-based) metrics, the normalized root mean square error was calculated [49]. The NRMSE is given by:

(13) $$NRMSE=\frac{RMSE}{max\left(x_{PPG}\right)-min\left(x_{PPG}\right)}$$

with

(14) $$RMSE=\sqrt{mean[{\left(x_{iPPG(i)}-x_{PPG(i)}\right)}^{2}]}$$

The normalization of the RMSE facilitated the performance comparison between the different extraction methods.

The Bland–Altman analysis was used to assess the agreement between the datasets made by the feature values for both iPPG and finger clip PPG methods. The coordinates of each point in the Bland–Altman plot were represented by the mean of the measurements using the iPPG signal, $x_{iPPG}$, and the finger clip PPG signal, $x_{PPG}$, per subject on the x-axis, as well as the difference between these two measurements on the y-axis:

(15) $$S\left(x_{iPPG},x_{PPG}\right)=\left(\frac{x_{iPPG}+x_{PPG}}{2},x_{iPPG}-x_{PPG}\right)$$

Bland–Altman quantifies the bias between the mean differences labeled as 95% limits of agreement (LoA). The LoA was calculated as follows:

(16) $$LoA\left(95\%\right)=\bar{d}\pm 1.96SD$$

where $\bar{d}$ is the mean difference between the iPPG-based estimation and the finger clip PPG-based reference value, and SD is the standard deviation of the differences [50].

## 3. Results

For all the different iPPG signal extraction methods, including RGD, AGRD, PCA, LE, SPE, ICA, CHROM, and POS (see Section 2.2.3), we extracted the PRV features listed in Table 2. For each of these methods, we employed the Spearman correlation coefficient (ρ) with its *p*-value, the NRMSE, and the Bland–Altman analysis, including the mean bias and its 95% confidence intervals (expressed by $\bar{d}\pm 1.96SD$).

### 3.1. Spearman Correlation

Table 3 summarizes the results of the Spearman correlation analysis on the PRV features, showing the correlation coefficients (ρ) and their statistical significance (*p*-value) per each iPPG-extraction method. Considering the averaged PR, Spearman’s correlation coefficient was higher than 0.9 in five of eight methods (except for PCA, LE, and SPE). We also obtained strong monotonic relationships (ρ>0.7) with statistical significance (*p* < 0.05) for SDNN in the time domain, VLF-power and LF-power in the frequency domain, and for SD2 and SD1/SD2 in the nonlinear domain. The CHROM and POS methods strongly correlated over all the above-mentioned features with, respectively, ρ=0.972 and ρ=0.994 for PR, ρ=0.768 and ρ=0.818 for SDNN, ρ=0.935 and ρ=0.939 for VLF-power, ρ=0.831 and ρ=0.903 for LF-power, ρ=0.882 and ρ=0.936 for SD2, and finally, ρ=0.734 and ρ=0.698 for SD1/SD2 (*p*-value < 0.001 for all).

### 3.2. Normalized Root Mean Square Error

Table 4 summarizes the results of the calculated values of NRMSE per iPPG extraction method for all subjects. When the NRMSE approaches the zero value, the iPPG-based extraction method fits the ground truth. Once the mean NRMSE < 0.20, the method was considered a good fit. For all features, high performance was obtained, except for the HF-power, as well as LF/HF in the frequency domain.

Concerning the time-domain analysis, the POS approach resulted to be the most performing, returning the lowest NRMSEs for all the features, except for the RMSSD values, which were better fitted by using GRD method. Moving to the frequency domain, the NRMSEs were higher than in the time domain. Moreover, in this case, the most promising results were obtained through the POS approach, with NRMSE < 0.20 for VLF-power, LF-power, HFnu, and LFnu. Interestingly, observing the results of the Poincaré map features (SD1, SD2, and SD1/SD2), a good performance of the GRD and POS methods can be noticed.

### 3.3. Bland–Altman Analysis

Bland–Altman analysis was applied to quantify the agreement between the PRV parameters derived from iPPG and finger clip PPG [51]. In Table 5, the Bland–Altman mean differences, $\bar{d}$, and the confident intervals, $\left[\bar{d}\pm 1.96SD\right]$, are depicted.

**Figure 4:**
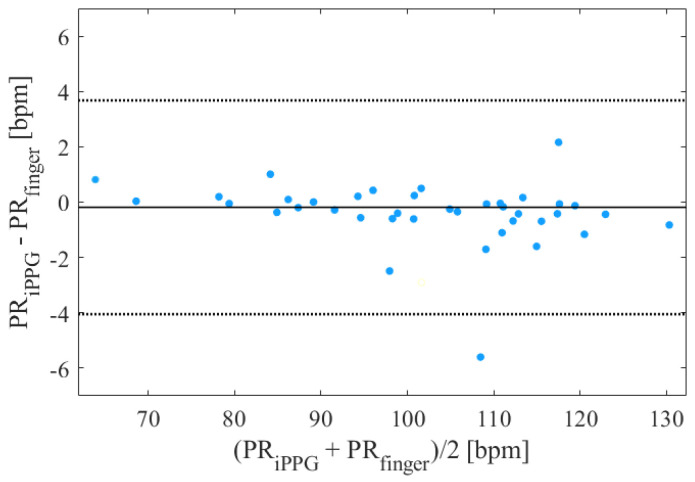
Bland−Altman plot of the averaged PR values obtained from iPPG extracted through the POS method and the finger clip PPG.

Table 5 reveals a close agreement between the iPPG- and PPG-based PRV feature extractions, especially in the time domain and for nonlinear features. Considering the PR, the LE method showed the smallest mean difference ($\bar{d}$ = −0.0409 BPM), but the corresponding 95% intervals reported a large variation between the estimations. On the other hand, the POS method showed more robustness with a mean difference of −0.186 BPM and a corresponding 95% confidence interval from −4.05 to 3.68 BPM (the corresponding Bland–Altman plot is reported in Figure 4). Regarding RMSSD, the GRD method disclosed the smallest mean bias and 95% confidence interval. Beholding SDNN, TI, and TINN, the CHROM method showed the smallest mean biases. However, the POS method, even if presenting higher $\bar{d}$ values, reduced the corresponding variations in the 95% limits. Based on the results of the Bland–Altman analysis, the POS and CHROM methods were found to also be the best for frequency features, except for VLF-power, where the lowest mean difference and confidence interval were found by applying the ICA method. Concerning the nonlinear techniques, the GRD method showed the highest agreement for SD1, with a mean bias of −2.37 ms and a corresponding 95% confidence interval from −31.6 to 26.8 ms. Regarding SD2, the POS method showed the highest accuracy with a mean bias of −2.47 ms and with 95% limits of agreement: −19.1 to 14.2 ms. As for the ratio of SD1 to SD2, the smallest biases were found using the GRD and POS methods (−0.163 and −0.169, respectively), but the narrowest confidence interval was obtained with the CHROM approach (from −0.491 to 0.136), implying more robustness. The CHROM method showed a corresponding mean bias of −0.177 for SD1/SD2.

## 4. Discussion and Conclusions

We evaluated contactless iPPG performance, comparing it with finger clip PPG, which is considered the ground truth for wearable monitoring [52,53]. The publicly available UBFC-RPPG dataset, including 42 subjects, was used to facilitate the replication of the results. The realistic human–machine interaction mimicked in the UBFC-RPPG dataset is a big step toward daily use of the contactless iPPG, as face motion (rigid and nonrigid), illumination variances, and subjects with make-up are included. The previous literature in the field of remote PPG usually presents a validation of the PPG signal extraction methods considering only the average of the PR. This procedure turns out to be incomplete and misleading, since, in this way, the artifacts are averaged out, resulting in an overall better agreement. A recent study compared the PRV extracted from iPPG with the HRV obtained from ECG during a specific experimental protocol in a controlled setting, looking at the reliability of the PRV features [50]. In this study, we extracted the PRV signals from the UBFC-RPPG videos, and we investigated the reliability of PRV features by comparing them with the corresponding wearable PPG values. We analyzed the PRV signals in both the time and frequency domains [2], together with the parameters extracted trough the Poincaré first return map, a technique taken from the theory of nonlinear time series analysis [46,47].

To investigate the feasibility of contactless iPPG-based PRV feature extraction in realistic daily settings, the iPPG extraction procedure was made more robust for the motion and illumination artifacts by applying ad hoc pre- and postprocessing steps, as described in Section 2.2.2 and Section 2.2.4. Eight different methods for iPPG signal extraction were used (see Section 2.2.3 for details): GRD, AGRD, PCA, LE, SPE, ICA, CHROM, and POS. These approaches included both linear (e.g., GRD) and nonlinear (e.g., LE) techniques already proposed in the past literature, but also other methodologies used here for the first time to process RGB images (i.e., SPE).

To evaluate the agreement of the contactless iPPG- and wearable PPG-based PRV features, we computed three statistical analyses: the Spearman correlation test, the NRMSE calculation, and the Bland–Altman analysis. Considering the results obtained from the three statistical analyses (see Table 3, Table 4 and Table 5), we were able to reach high performances in the computation of PR mean, especially with the GRD, AGRD, ICA, CHROM, and POS methods. Looking at the standard PRV features, the POS method resulted to be the most reliable in both time and frequency domain, with a higher number of features with the biggest correlation coefficients, the lowest NRMSEs, and the narrowest confidence intervals in the Bland–Altman analysis. It showed the best results for the features which are related to the overall autonomic activity (e.g., SDNN, TINN, VLF-power, and LF-power), without specific physiological correlates in terms of sympathetic and parasympathetic modulation. However, the real power of HRV and PRV, as tools for cardiovascular assessment, is given by the strong association between vagal activity and specific features related to short-term variability of such time series, for example RMSSD and HF-power-related features. Analyzing the values of the normalized HF-power (HF-power nu), we noticed an acceptable level of agreement (NRMSE = 0.19), a low bias ($\bar{d}=-1.26$), and a significant correlation between iPPG and wearable PPG, but with a correlation coefficient equal to 0.49.

The study of the Poincaré map allowed us to broaden the investigation on the reliability of iPPG as a technique for the assessment of autonomic regulation. In fact, by adding to the list of standard features those calculated from the shape of the Poincaré plot using the ellipse fitting method, it is possible to evaluate two other parameters traditionally linked to vagal activity: SD1 and SD1/SD2 [54,55]. As for SD1, the most promising findings were obtained by applying the GRD method, but the Spearman correlation coefficient was equal to 0.4. The CHROM and POS methods outperformed the other methods for SD2 and SD12 computation. The iPPG SD1/SD2 was demonstrated to be the vagal-correlated parameter with the highest reliability, showing a Spearman correlation coefficient of 0.7, and of 0.73 when the POS and CHROM methods were used.

Our results suggest the use of the POS and CHROM techniques for the extraction of the PPG signal from ultrashort videos, since the related PRV features are the most reliable when compared with those obtained with wearable devices. In fact, using these approaches, it was proven that it is possible to characterize both overall autonomic (by computing SDNN or LF-power) and specific vagal dynamics (by extracting SD1/SD2). Approaches based on nonlinear techniques for dimensionality reduction (LE and SPE) led to less promising results. This poor performance could be due to the short duration of RGB signals (approximately 1 min 8 s).

To conclude, we can state that an iPPG system can successfully provide clinically useful information about autonomic dynamics, even when ultrashort video clips (less than 5 min [53,56]) are recorded. The possibility of extracting reliable information regarding changes in vagal activity through the Poincaré analysis of PRV signals obtained from iPPG techniques would allow to monitor the progress of many pathologies in a completely contactless manner. It is known, in fact, that reduced vagal activity correlates with various cardiovascular diseases, including heart failure, ischemia injury, arrhythmia, and hypertension [57], but also with metabolic [58] and mental disorders [59]. The remarkable agreement found for the SD1/SD2 feature might justify and promote the use of nonlinear methods, and especially the Poincaré map, as a reliable tool for iPPG analysis and vagal activity investigation. Cardiovascular assessment through common, cheap, and contactless devices would significantly improve the possibilities and conditions of monitoring, for example, in home monitoring applications, in periods when physical contact is not recommended (possible pandemics), and in subjects for whom even wearable devices are too obtrusive (infants and newborns).

## 5. Limitations and Future Work

The main limitations of our study concern the RGB signal extraction. In our study, the whole face was chosen as ROI. To automatize the ROI detection the Viola–Jones (face detection) and KLT tracker (face tracking) algorithms were applied. The downside hereof is that the face must always be visible, otherwise, the algorithm fails to find the ROI. Moreover, to create the skin mask, a simple thresholding method based on an empirical skin color distribution (RGBHH-CbCr model) was applied. However, when, for example, hair falls in the same color range, it is not excluded from the skin mask, disturbing the PPG extraction. In future works, making use of adaptive skin segmentation might benefit the quality of the RGB signal [27,60].

Our future research in the field of rPPG will also be directed towards the investigation of the influence of the recording length on the performance of nonlinear embedding size reduction techniques, such as LE or the here-proposed SPE. Indeed, considering that in previous works the LE method had been declared to be the most promising technique [14], we hypothesize that the recoding length could be a probable cause of the poor performance we found. On the other hand, considering that the CHROM and POS methods resulted to be the most performing with the UBFC-RPPG dataset, our future analyses will be oriented towards the application of these methods to other datasets with different light conditions and duration, as well as towards the optimization of these algorithms to test whether the introduction of specific changes could further improve their performance. Moreover, in future studies, we will always test the reliability of existing and new algorithms as a function of the quality of the videos, using the algorithms for image quality assessment present in the literature [25,26,61].

## Figures and Tables

**Table 1:** Brief description of the eight methodologies used for iPPG extraction.

iPPG Extraction Methods	Short Description
Green–Red Difference (GRD)	iPPG is estimated by the green signal, while the red signal is considered as containing artifacts [17]
Adaptive Green–Red Difference (AGRD)	An adaptive color difference operation between the green and red channels is applied to reduce motion artifacts [18]
Principal Component Analysis (PCA)	The most relevant information of RGB data is expressed as a set of new orthogonal variables, called principal components [19]
Independent Component Analysis (ICA)	RGB signals are decomposed by means of blind source separation and the component with the most prominent peak in the PR bandwith is chosen according to [22]
Chrominace-Based (CHROM)	A model of PPG-induced variations in color intensities is employed to improve motion robustness [23]
Plane-Orthogonal-to-Skin (POS)	Improved version of CHROM method which uses a projection plane orthogonal to the skin tone for pulse extraction [13]
Laplacian Eigenmap (LE)	Unfolds data distribution in a hyperdimensional space in order to reduce dimensionality [20]
Stochastic Proximity Embedding (SPE)	Generates an one-dimensional Euclidean embedding out of the RGB-data, where the similarities between the related observations are preserved [21]

**Table 2:** PRV metrics in the time, frequency, and nonlinear domains.

PRV Metrics	Unit	Description
**Time domain**		
PR	1/min	Average pulse rate
RMSSD	ms	Root mean square of successive IBI interval differences
SDNN	ms	Standard deviation of NN intervals
TI	-	Integral of NN interval histogram divided by its height
TINN	ms	Baseline width of the NN interval histogram
**Frequency domain**		
VLF	ms^2^	Absolute power of the very-low-frequency band (0.0033–0.04 Hz)
LF	ms^2^	Absolute power of the low-frequency power (0.04–0.15 Hz)
HF	ms^2^	High-frequency power (0.15–0.4 Hz)
LF/HF	-	Ratio of LF-to-HF absolute power
LFnu	n.u.	Relative power in the low-frequency band
HFnu	n.u.	Relative power in the high-frequency band in normal units
**Nonlinear domain**		
SD1	ms	Poincaré plot standard deviation perpendicular to the line of identity
SD2	ms	Poincaré plot standard deviation along the line of identity
SD1/SD2	-	Ratio of SD1-to-SD2 standard deviations

**Table 3:** Spearman’s correlation coefficients and corresponding *p*-values calculated between the values of PRV features extracted through the eight iPPG methods and with the wearable sensor. Bold indicates the lowest NRMSE for each PRV feature.

Spearman Correlation Coefficient (ρ) and Statistical Significance (*p*-Value)									
		**GRD**	**AGRD**	**PCA**	**LE**	**SPE**	**ICA**	**CHROM**	**POS**
**Time domain**									
PR	ρ *p*-val	0.980 $1.48\times 10^{-29}$	0.957 $3.76\times 10^{-23}$	0.583 $5.03\times 10^{-5}$	0.437 0.004	0.336 0.03	0.916 $2.05\times 10^{-17}$	0.971 $1.61\times 10^{-26}$	**0.994** ** $2.61\times 10^{-40}$ **
RMSSD	ρ *p*-val	**0.376** ** $1.40\times 10^{-2}$ **	0.330 0.03	−0.048 0.76	−0.009 0.95	0.168 0.23	0.253 0.11	**0.376** **0.01**	0.374 0.01
SDNN	ρ *p*-val	0.601 $2.56\times 10^{-5}$	0.585 $4.77\times 10^{-5}$	0.278 0.07	−0.017 0.92	0.336 0.03	0.676 $9.20\times 10^{-7}$	0.768 $3.01\times 10^{-9}$	**0.818** ** $3.63\times 10^{-11}$ **
TI	ρ *p*-val	0.477 $1.41\times 10^{-3}$	0.471 $1.65\times 10^{-3}$	0.202 0.20	0.147 0.15	0.142 0.37	0.530 $3.09\times 10^{-4}$	**0.623** ** $1.05\times 10^{-5}$ **	0.592 $3.64\times 10^{-5}$
TINN	ρ *p*-val	0.340 $2.74\times 10^{-2}$	0.331 $3.21\times 10^{-2}$	−0.085 0.59	−0.153 0.33	0.167 0.37	0.416 $6.15\times 10^{-3}$	0.482 $1.23\times 10^{-3}$	**0.510** ** $5.63\times 10^{-4}$ **
**Frequency domain**									
VLF-pow	ρ *p*-val	0.719 $8.10\times 10^{-8}$	0.687 $5.13\times 10^{-7}$	0.021 0.90	−0.113 0.47	0.192 0.22	0.915 $2.55\times 10^{-17}$	0.935 $1.13\times 10^{-19}$	**0.939** ** $3.75\times 10^{-20}$ **
LF-pow	ρ *p*-val	0.391 $3.91\times 10^{-1}$	0.356 0.02	0.267 0.09	−0.043 0.79	0.126 0.43	0.659 $2.12\times 10^{-6}$	0.831 $9.22\times 10^{-12}$	**0.903** ** $2.69\times 10^{-16}$ **
HF-pow	ρ *p*-val	0.064 0.69	−0.040 0.80	−0.102 0.52	−0.096 0.55	−0.103 0.52	−0.09 0.57	**0.348** **0.02**	0.134 0.40
LF/HF	ρ *p*-val	−0.222 0.16	−0.0925 0.56	0.412 $6.78\times 10^{-3}$	0.025 0.88	0.178 0.26	−0.098 0.54	**−0.313** **0.04**	−0.192 0.23
HFnu	ρ *p*-val	0.340 0.03	0.142 0.37	0.035 0.82	−0.179 0.26	0.028 0.86	0.286 0.07	0.478 $1.38\times 10^{-3}$	**0.493** ** $9.09\times 10^{-4}$ **
LFnu	ρ *p*-val	0.325 0.04	0.134 0.40	0.040 0.80	−0.170 0.28	0.029 0.86	0.278 0.07	0.477 $1.42\times 10^{-3}$	**0.499** ** $7.75\times 10^{-4}$ **
**Nonlinear domain**									
SD1	ρ *p*-val	**0.384** ** $1.19\times 10^{-2}$ **	0.330 0.03	−0.048 0.76	−0.013 0.93	0.160 0.31	0.252 0.11	0.376 0.01	0.379 0.01
SD2	ρ *p*-val	0.687 $5.00\times 10^{-7}$	0.664 $1.67\times 10^{-6}$	0.327 0.03	−0.072 0.65	0.365 0.02	0.816 $4.55\times 10^{-11}$	0.882 $1.17\times 10^{-14}$	**0.936** ** $8.85\times 10^{-20}$ **
SD1/SD2	ρ *p*-val	0.632 $7.12\times 10^{-6}$	0.588 $4.30\times 10^{-5}$	0.032 0.84	−0.136 0.39	0.100 0.53	0.689 $4.62\times 10^{-7}$	**0.734** ** $3.28\times 10^{-8}$ **	0.698 $2.74\times 10^{-7}$

**Table 4:** Normalizedroot mean square errors (NRMSE) calculated between the values of PRV features extracted through the eight iPPG methods and with the wearable sensor. Bold indicates the best performance in the correlation tests for each PRV feature.

Normalized Root Mean Square Error (NRMSE)								
	**GRD**	**AGRD**	**PCA**	**LE**	**SPE**	**ICA**	**CHROM**	**POS**
**Time domain**								
PR	0.0393	0.0466	0.158	0.182	0.243	0.0348	0.0278	**0.014**
RMSSD	**0.179**	0.197	0.380	0.392	0.379	0.225	0.221	0.228
SDNN	0.176	0.189	0.587	0.726	0.605	0.148	0.144	**0.099**
TI	0.210	0.226	0.671	0.676	0.720	0.194	0.178	**0.163**
TINN	0.261	0.237	0.553	0.724	0.750	0.200	0.222	**0.172**
**Frequency domain**								
VLF-power	0.152	0.112	0.518	0.473	0.216	0.0485	0.064	**0.038**
LF-power	0.243	0.273	1.42	1.46	1.34	0.168	0.166	**0.061**
HF-power	0.988	1.03	1.06	1.09	1.05	1.03	**0.927**	0.928
LF/HF	0.232	0.224	**0.215**	0.218	0.241	0.229	0.234	0.230
HFnu	0.231	0.261	0.336	0.330	0.286	0.244	0.203	**0.194**
LFnu	0.231	0.260	0.331	0.328	0.283	0.243	0.202	**0.193**
**Nonlinear domain**								
SD1	**0.179**	0.196	0.378	0.389	0.376	0.223	0.220	0.227
SD2	0.194	0.215	0.644	0.821	0.675	0.135	0.129	**0.063**
SD1/SD2	0.202	0.207	0.245	0.298	0.270	0.192	0.205	**0.191**

**Table 5:** Bland–Altman Analysis results in terms of mean differences $\bar{d}$ and 95% confidence intervals $\left[\bar{d}\pm 1.96SD\right]$ for each iPPG method when compared with the ground truth.

Bland–Altman Analysis								
	**GRD**	**AGRD**	**PCA**	**LE**	**SPE**	**ICA**	**CHROM**	**POS**
**Time domain**								
PR	−0.474	−1.14	−4.53	−0.041	13.2	−1.90	1.05	**−0.186**
[1/min]	[−9.81,8.86]	[−12.7,10.4]	[−34.7,25.6]	[−34.2,34.1]	[−28.9,55.3]	[−14.0,10.2]	[−9.62,11.7]	**[−4.05,3.68]**
RMSSD	**−3.37**	−5.97	29.4	31.9	29.7	−8.89	−13.5	−17.3
[ms]	**[−44.3,37.6]**	[−49.3,37.4]	[−42.7,102]	[−29,92.8]	[−18.3,77.8]	[−56.6,38.8]	[−54.8,27.9]	[−58.4,23.8]
SDNN	8.65	8.99	47.6	59.6	47.8	1.55	1.11	**−6.18**
[ms]	[−30.3,47.6]	[−30.1,48.1]	[−33,128]	[−35.8,155]	[−21.6,117]	[−32.3,35.4]	[−42.2,44.4]	**[−25.5,13.1]**
TI	0.374	0.465	2.57	2.91	2.97	0.210	0.149	**−0.208**
	[−2.10,2.85]	[−2.07,3.00]	[−2.21,7.34]	[−1.28,7.10]	[−0.16,6.09]	[−2.25,2.67]	[−1.84,2.14]	**[−1.72,1.30]**
TINN	0.035	0.030	0.147	0.198	0.180	0.004	0.002	**−0.024**
[ms]	[−0.14,0.21]	[−0.12,0.18]	[−0.11,0.40]	[−0.08,0.48]	[−0.07,0.43]	[−0.14,0.15]	[−0.18,0.18]	**[−0.13,0.08]**
**Frequency domain**								
VLFpow	161 × 10	511	207 × 10	443 × 10	147 × 10	**-200**	554	−270
[ms${}^{2}$]	[−176,208]$\;\times \;10^{2}$	[−296,398] × 10	[−60.1,101]$\;\times \;10^{2}$	[128,216]$\;\times \;10^{2}$	[−80.0,109]$\;\times \;10^{2}$	**[−221,181] × 10**	[−566,677] × 10	[−304,250] × 10
LFpow	597	792	510 × 10	514 × 10	454 × 10	308	452	**128**
[ms${}^{2}$]	[−204,323] × 10	[−228,387] × 10	[−120,212]$\;\times \;10^{2}$	[−88.4,191]$\;\times \;10^{2}$	[−214,305]$\;\times \;10^{2}$	[−175,236] × 10	[−197,288] × 10	**[−525,782]**
HFpow	−243$\;\times \;10^{3}$	−254$\;\times \;10^{3}$	−261$\;\times \;10^{3}$	−268$\;\times \;10^{3}$	−259$\;\times \;10^{3}$	−254$\;\times \;10^{3}$	**−228$\;\times \;10^{3}$**	−228$\;\times \;10^{3}$
[ms${}^{2}$]	[−433,−53.7]$\;\times \;10^{3}$	[−449,−60]$\;\times \;10^{3}$	[−456,21.2]$\;\times \;10^{3}$	[−462,−74.1]$\;\times \;10^{3}$	[−457,−60.3]$\;\times \;10^{3}$	[−444,−63.2]$\;\times \;10^{3}$	**[−408,−47.7]$\;\times \;10^{3}$**	[−409,−48]$\;\times \;10^{3}$
LF/HF	−1.47	−1.44	−1.57	−1.40	−1.69	−1.47	**−1.41**	−1.45
	[−4.40,1.47]	[−4.48,1.60]	[−5.09,1.96]	[−4.27,1.46]	[−5.32,1.94]	[−4.35,1.40]	**[−4.07,1.25]**	[−4.30,1.40]
HFnu	4.65	1.08	1.20	−3.18	3.58	3.87	−0.472	**−1.26**
[n.u.]	[−36.0,45.3]	[−47.0,49.1]	[−55.8,58.2]	[−62.7,56.4]	[−47.7,54.8]	[−37.9,45.7]	[−36.0,35.1]	**[−35.7,33.1]**
LFnu	−4.60	−0.953	−0.984	3.39	−3.41	−3.73	**0.545**	**1.31**
[n.u.]	[−45.7,36.5]	[−49.2,47.3]	[−58.0,56.1]	[−56.5,63.2]	[−54.8,48.0]	[−45.7,38.2]	**[−35.2,36.3]**	**[−33.2,35.9]**
**Nonlinear domain**								
SD1	**−2.37**	−4.25	21.1	22.8	21.1	−6.35	−9.58	−12.3
[ms]	**[−31.6,26.8]**	[−35.2,26.7]	[−30.7,72.9]	[−21.0,66.6]	[−13.4,55.7]	[−40.4,27.7]	[−39.1,19.9]	[−41.7,17.0]
SD2	15.9	17.9	67.1	85.0	67.7	7.33	7.42	**−2.47**
[ms]	[−36.7,68.5]	[−34.6,70.5]	[−47.5,182]	[−56.2,226]	[−35.2,171]	[−32.3,46.9]	[−51.9,66.7]	**[−19.1,14.2]**
SD1/SD2	−0.163	−0.207	−0.199	−0.243	-0.238	−0.169	**−0.177**	−0.169
	[−0.58,0.26]	[−0.59,0.18]	[−0.78,0.38]	[−0.85,0.37]	[−0.80,0.33]	[−0.52,0.18]	**[−0.49,0.14]**	[−0.49,0.16]

## Data Availability

The data presented in this study are openly available at https://sites.google.com/view/ybenezeth/ubfcrppg?pli=1 (accessed on 5 December 2022).

## Abbreviations

The following abbreviations are used in this manuscript:

AGRD	Adaptive green–red difference
ANS	Autonomic nervous system
CHROME	Chrominace-based
GRD	Green–red difference
HR	Heart rate
HRV	Heart rate variability
IBI	Interbeat intervals
ICA	Independent component analysis
iPPG	Image-based photoplethysmography
LE	Laplacian eigenmap
PCA	Principal component analysis
POS	Plane-orthogonal-to-skin
PPG	Photoplethysmography
PR	Pulse rate
PRV	Pulse rate variability
RGB	Red-green-blue
ROI	Region of interest
SPE	Stochastic proximity embedding

## References

[1] Favilla R., Zuccala V.C., Coppini G. (2019). Heart Rate and Heart Rate Variability from Single-Channel Video and ICA Integration of Multiple Signals. IEEE J. Biomed. Health Inform..

[2] Acharya U.R., Joseph K.P., Kannathal N., Lim C.M., Suri J.S. (2006). Heart rate variability: A review. Med. Biol. Eng. Comput..

[3] Gil E., Orini M., Bailón R., Vergara J.M., Mainardi L., Laguna P. (2010). Photoplethysmography pulse rate variability as a surrogate measurement of heart rate variability during non-stationary conditions. Physiol. Meas..

[4] Yu S.G., Kim S.E., Kim N.H., Suh K.H., Lee E.C. (2021). Pulse rate variability analysis using remote photoplethysmography signals. Sensors.

[5] Monkaresi H., Bosch N., Calvo R.A., D’Mello S.K. (2017). Automated Detection of Engagement Using Video-Based Estimation of Facial Expressions and Heart Rate. IEEE Trans. Affect. Comput..

[6] Sigari M.H., Fathy M., Soryani M. (2013). A driver face monitoring system for fatigue and distraction detection. Int. J. Veh. Technol..

[7] Sasangohar F., Davis E., Kash B.A., Shah S.R. (2018). Remote Patient Monitoring and Telemedicine in Neonatal and Pediatric Settings: Scoping Literature Review. J. Med. Internet Res..

[8] Garbey M., Sun N., Merla A., Pavlidis I. (2007). Contact-free measurement of cardiac pulse based on the analysis of thermal imagery. IEEE Trans. Biomed. Eng..

[9] Boric-Lubecke O., Lubecke V., Mostafanezhad I. Amplitude modulation issues in Doppler radar heart signal extraction. Proceedings of the 2011 IEEE Radio and Wireless Week, RWW 2011—2011 IEEE Topical Conference on Biomedical Wireless Technologies, Networks, and Sensing Systems, BioWireleSS 2011.

[10] Mesleh A., Skopin D., Baglikov S., Quteishat A. (2012). Heart Rate Extraction from Vowel Speech Signals. J. Comput. Sci. Technol..

[11] Poh M.Z., McDuff D.J., Picard R.W. (2011). Advancements in noncontact, multiparameter physiological measurements using a webcam. IEEE Trans. Bio-Med. Eng..

[12] Pursche T., Krajewski J., Moeller R. Video-based heart rate measurement from human faces. Proceedings of the 2012 IEEE International Conference on Consumer Electronics (ICCE).

[13] Wang W., Den Brinker A.C., Stuijk S., De Haan G. (2017). Algorithmic Principles of Remote PPG. IEEE Trans. Biomed. Eng..

[14] Sikdar A., Behera S.K., Dogra D.P. (2016). Computer-vision-guided human pulse rate estimation: A review. IEEE Rev. Biomed. Eng..

[15] Unakafov A.M. (2018). Pulse rate estimation using imaging photoplethysmography: Generic framework and comparison of methods on a publicly available dataset. Biomed. Phys. Eng. Express.

[16] Sun Y., Thakor N. (2016). Photoplethysmography Revisited: From Contact to Noncontact, from Point to Imaging. IEEE Trans. Biomed. Eng..

[17] Hülsbusch M. (2008). An Image-Based Functional Method for Opto-Electronic Detection of Skin-Perfusion.

[18] Feng L., Po L.M., Xu X., Li Y., Ma R. (2015). Motion-resistant remote imaging photoplethysmography based on the optical properties of skin. IEEE Trans. Circuits Syst. Video Technol..

[19] Abdi H., Williams L.J. (2010). Principal component analysis. Wiley Interdiscip. Rev. Comput. Stat..

[20] Wei L., Tian Y., Wang Y., Ebrahimi T., Huang T. Automatic webcam-based human heart rate measurements using laplacian eigenmap. Proceedings of the Computer Vision–ACCV 2012: 11th Asian Conference on Computer Vision.

[21] Agrafiotis D.K. (2003). Stochastic proximity embedding. J. Comput. Chem..

[22] Cardoso J.F. (1999). High-Order Contrasts for Independent Component Analysis. Neural Comput..

[23] De Haan G., Jeanne V. (2013). Robust pulse rate from chrominance-based rPPG. IEEE Trans. Biomed. Eng..

[24] Bobbia S., Macwan R., Benezeth Y., Mansouri A., Dubois J. (2019). Unsupervised skin tissue segmentation for remote photoplethysmography. Pattern Recognit. Lett..

[25] Mittal A., Moorthy A.K., Bovik A.C. (2012). No-reference image quality assessment in the spatial domain. IEEE Trans. Image Process..

[26] Venkatanath N., Praneeth D., Bh M.C., Channappayya S.S., Medasani S.S. Blind image quality evaluation using perception based features. Proceedings of the 2015 Twenty First National Conference on Communications (NCC).

[27] Fouad R.M., Omer O.A., Aly M.H. (2019). Optimizing Remote Photoplethysmography Using Adaptive Skin Segmentation for Real-Time Heart Rate Monitoring. IEEE Access.

[28] Lewandowska M., Rumiński J., Kocejko T., Nowak J. Measuring pulse rate with a webcam—A non-contact method for evaluating cardiac activity. Proceedings of the 2011 Federated Conference on Computer Science and Information Systems (FedCSIS).

[29] Mannapperuma K., Holton B.D., Lesniewski P.J., Thomas J.C. (2014). Performance limits of ICA-based heart rate identification techniques in imaging photoplethysmography. Physiol. Meas..

[30] McDuff D., Gontarek S., Picard R. Remote measurement of cognitive stress via heart rate variability. Proceedings of the 2014 36th Annual International Conference of the IEEE Engineering in Medicine and Biology Society, EMBC 2014.

[31] Chen J., Patel V.M., Liu L., Kellokumpu V., Zhao G., Pietikäinen M., Chellappa R. (2017). Robust local features for remote face recognition. Image Vis. Comput..

[32] Verkruysse W., Svaasand L.O., Nelson J.S. (2008). Remote plethysmographic imaging using ambient light. Opt. Express.

[33] Viola P., Jones M. Rapid object detection using a boosted cascade of simple features. Proceedings of the 2001 IEEE Computer Society Conference on Computer Vision and Pattern Recognition, CVPR 2001.

[34] Baker S., Matthews I. (2004). Lucas-Kanade 20 Years On: A Unifying Framework. Int. J. Comput. Vis..

[35] Bin Abdul Rahman N.A., Wei K.C., See J. (2007). Rgb-h-cbcr Skin Colour Model for Human Face Detection.

[36] Holton B.D., Mannapperuma K., Lesniewski P.J., Thomas J.C. (2013). Signal recovery in imaging photoplethysmography. Physiol. Meas..

[37] Tarvainen M.P., Ranta-aho P.O., Karjalainen P.A. (2002). An advanced detrending method with application to HRV analysis. IEEE Trans. Biomed. Eng..

[38] Tarassenko L., Villarroel M., Guazzi A., Jorge J., Clifton D.A., Pugh C. (2014). Non-contact video-based vital sign monitoring using ambient light and auto-regressive models. Physiol. Meas..

[39] Jain M., Deb S., Subramanyam A.V. Face video based touchless blood pressure and heart rate estimation. Proceedings of the 2016 IEEE 18th International Workshop on Multimedia Signal Processing, MMSP 2016.

[40] Poh M.Z., McDuff D.J., Picard R.W. (2010). Non-contact, automated cardiac pulse measurements using video imaging and blind source separation. Opt. Express.

[41] Bousefsaf F., Maaoui C., Pruski A. (2016). Peripheral vasomotor activity assessment using a continuous wavelet analysis on webcam photoplethysmographic signals. Bio-Med. Mater. Eng..

[42] Wu B.F., Chu Y.W., Huang P.W., Chung M.L., Lin T.M. (2017). A motion robust remote-PPG approach to driver’s health state monitoring. Lect. Notes Comput. Sci. (Incl. Subser. Lect. Notes Artif. Intell. Lect. Notes Bioinform.).

[43] Schäfer A., Vagedes J. (2013). How accurate is pulse rate variability as an estimate of heart rate variability? A review on studies comparing photoplethysmographic technology with an electrocardiogram. Int. J. Cardiol..

[44] Elgendi M., Norton I., Brearley M., Abbott D., Schuurmans D. (2013). Systolic Peak Detection in Acceleration Photoplethysmograms Measured from Emergency Responders in Tropical Conditions. PLoS ONE.

[45] Tarvainen M.P., Niskanen J.P., Lipponen J.A., Ranta-Aho P.O., Karjalainen P.A. (2014). Kubios HRV–heart rate variability analysis software. Comput. Methods Programs Biomed..

[46] Tulppo M.P., Makikallio T.H., Takala T., Seppanen T., Huikuri H.V. (1996). Quantitative beat-to-beat analysis of heart rate dynamics during exercise. Am. J. Physiol.-Heart Circ. Physiol..

[47] Nardelli M., Greco A., Bolea J., Valenza G., Scilingo E.P., Bailon R. (2017). Reliability of lagged poincaré plot parameters in ultrashort heart rate variability series: Application on affective sounds. IEEE J. Biomed. Health Inform..

[48] Lilliefors H.W. (1967). On the Kolmogorov-Smirnov test for normality with mean and variance unknown. J. Am. Stat. Assoc..

[49] Zhang B., Sennrich R. (2019). Root mean square layer normalization. Adv. Neural Inf. Process. Syst..

[50] Shoushan M.M., Reyes B.A., Mejia Rodriguez A.R., Chong J.W. (2022). Contactless Monitoring of Heart Rate Variability During Respiratory Maneuvers. IEEE Sens. J..

[51] Breslow N.E. (2014). Lessons in biostatistics. Past Present Future Stat. Sci..

[52] Hartmann V., Liu H., Chen F., Qiu Q., Hughes S., Zheng D. (2019). Quantitative comparison of photoplethysmographic waveform characteristics: Effect of measurement site. Front. Physiol..

[53] Nardelli M., Vanello N., Galperti G., Greco A., Scilingo E.P. (2020). Assessing the quality of heart rate variability estimated from wrist and finger ppg: A novel approach based on cross-mapping method. Sensors.

[54] Park S.K., Kang S.J., Im H.S., Cheon M.Y., Bang J.Y., Shin W.J., Choi B.M., Youn M.O., Kim Y.K., Hwang G.S. (2005). Validity of Heart Rate Variability Using Poincare Plot for Assessing Vagal Tone during General Anesthesia. Korean J. Anesthesiol..

[55] Keute M., Machetanz K., Berelidze L., Guggenberger R., Gharabaghi A. (2021). Neuro-cardiac coupling predicts transcutaneous auricular vagus nerve stimulation effects. Brain Stimul..

[56] Shaffer F., Meehan Z.M., Zerr C.L. (2020). A critical review of ultra-short-term heart rate variability norms research. Front. Neurosci..

[57] He X., Zhao M., Bi X., Sun L., Yu X., Zhao M., Zang W. (2015). Novel strategies and underlying protective mechanisms of modulation of vagal activity in cardiovascular diseases. Br. J. Pharmacol..

[58] Dangardt F., Volkmann R., Chen Y., Osika W., Mårild S., Friberg P. (2011). Reduced cardiac vagal activity in obese children and adolescents. Clin. Physiol. Funct. Imaging.

[59] Clamor A., Lincoln T.M., Thayer J.F., Koenig J. (2016). Resting vagal activity in schizophrenia: Meta-analysis of heart rate variability as a potential endophenotype. Br. J. Psychiatry.

[60] Phung S.L., Chai D., Bouzerdoum A. Adaptive skin segmentation in color images. Proceedings of the 2003 IEEE International Conference on Acoustics, Speech, and Signal Processing, Proceedings (ICASSP’03).

[61] Niu Y., Zhong Y., Guo W., Shi Y., Chen P. (2018). 2D and 3D image quality assessment: A survey of metrics and challenges. IEEE Access.

